# Activity of benzimidazoles against *Dientamoeba fragilis* (Trichomonadida, Monocercomonadidae) *in vitro* and correlation of beta-tubulin sequences as an indicator of resistance

**DOI:** 10.1051/parasite/2014043

**Published:** 2014-08-25

**Authors:** Damien Stark, Joel L.N. Barratt, Tamalee Roberts, Deborah Marriott, John T. Harkness, John Ellis

**Affiliations:** 1 Division of Microbiology, SydPath, St. Vincent’s Hospital Darlinghurst NSW 2010 Australia; 2 University of Technology Sydney, School of Medical and Molecular Biosciences Broadway 2007 Australia; 3 University of Technology Sydney, iThree Institute Broadway 2007 Australia

**Keywords:** *Dientamoeba fragilis*, Antimicrobials, Benzimidazoles, Beta-tubulin

## Abstract

Recently, *Dientamoeba fragilis* has emerged as a significant and common enteropathogen. The majority of patients with dientamoebiasis present with gastrointestinal complaints and chronic symptoms are common. Numerous studies have successfully demonstrated parasite clearance, coupled with complete resolution of clinical symptoms following treatment with various antiparasitic compounds. Despite this, there is very little *in vitro* susceptibility data available for the organism. Benzimidazoles are a class of antiparasitic drugs that are commonly used for the treatment of protozoan and helminthic infections. Susceptibility testing was undertaken on four *D. fragilis* clinical isolates against the following benzimidazoles: albendazole, flubendazole, mebendazole, nocodazole, triclabendazole and thiabendazole. The activities of the antiprotozoal compounds at concentrations ranging from 2 μg/mL to 500 μg/mL were determined via cell counts of *D. fragilis* grown in xenic culture. All tested drugs showed no efficacy. The beta-tubulin transcript was sequenced from two of the *D. fragilis* isolates and amino acid sequences predicted a susceptibility to benzimidazoles. This is the first study to report susceptibility profiles for benzimidazoles against *D. fragilis*, all of which were not active against the organism. This study also found that beta-tubulin sequences cannot be used as a reliable marker for resistance of benzimidazoles in *D. fragilis*.

## Abstract (fr)

Récemment, *D. fragilis* a émergé comme un entéropathogène important et commun. La majorité des patients avec dientamoebiase présente des troubles gastro-intestinaux et les symptômes chroniques sont fréquents. De nombreuses études ont démontré avec succès l’élimination des parasites, couplée à la résolution complète des symptômes cliniques, après traitement avec divers composés antiparasitaires. Malgré cela, il y a très peu de données disponibles sur la sensibilité *in vitro* de cet organisme. Les benzimidazoles sont une classe de médicaments antiparasitaires qui sont couramment utilisés pour le traitement des infections à protozoaires et helminthes. Les tests de sensibilité ont été réalisés sur quatre isolats cliniques de *D. fragilis* avec les benzimidazoles suivants : albendazole, flubendazole, mébendazole, nocodazole, triclabendazole et thiabendazole. Les activités des composés antiprotozoaires, à des concentrations allant de 2 μg/ml à 500 μg/ml ont été déterminées par comptage de cellules de *D. fragilis* cultivées en culture xénique. Tous les médicaments testés n’ont montré aucune efficacité. Le transcript de béta-tubuline a été séquencé à partir de deux isolats de *D. fragilis*, et les séquences d’acides aminés prédisaient une sensibilité aux benzimidazoles. Cette étude est la première à signaler des profils de sensibilité pour les benzimidazoles contre *D. fragilis*, qui tous étaient non actifs contre l’organisme. Cette étude a également révélé que les séquences de béta-tubuline ne peuvent pas être utilisées comme un marqueur fiable de la résistance de benzimidazoles chez *D. fragilis*.

## Introduction

*Dientamoeba fragilis* Jepps and Dobell, 1918 [18] is a protozoan parasite that is the only recognised species in the genus *Dientamoeba*. It is classified as a trichomonad in the class Trichomonadida and has been shown to be closely related to the amoeboflagellate *Histomonas meleagridis* [14]. *Dientamoeba* is emerging as one of the most commonly encountered enteric protozoa of humans with prevalence reaching up to 43% in some studies when appropriate diagnostic methods are utilised [27]. Despite this, it continues to be neglected as a significant pathogen, with many laboratories not routinely performing adequate laboratory diagnostic testing for the parasite [2, 7, 12].

The clinical presentation of dientamoebiasis varies from asymptomatic carriage to symptomatic presentations, ranging from altered bowel motions, abdominal discomfort, nausea, and diarrhoea [28, 29, 33, 35]. The propensity of the organism to cause chronic symptoms, ranging from weeks to months, has been reported in the scientific literature [7, 15]. The life cycle and mode of transmission of *D. fragilis* are poorly defined. However, the recent discovery of a cyst stage in the life cycle of this parasite would suggest that direct transmission via the faecal-oral route is the most likely mode of transmission [24]. High rates of transmission between close contacts and household members have been described, highlighting the transmissible nature of the organism [31].

Despite the discovery of the parasite nearly 100 years ago and the abundance of reports in the scientific literature regarding infections, very little research has been conducted on the use of suitable antimicrobial compounds to control infections and subsequent susceptibility testing of isolates [32]. Only three studies to date have undertaken *in vitro* susceptibility testing on *D. fragilis* isolates [3, 10, 25], and no studies to date have looked at the efficacy of the benzimidazoles. Benzimidazoles have been shown to be effective in treating both *Trichomonas vaginalis* [20, 21] and *Giardia intestinalis* [38] and ineffective against *H. meleagridis* [9, 17]. Benzimidazoles are a class of antiparasitic drug [5], which act on beta-tubulin by binding to a high-affinity binding site on the beta-tubulin monomer [22]. There are several different beta-tubulin residues that have been proposed as indicators of benzimidazole susceptibility. In protozoa, two residues, Glu-198 and Phe-200, have been hypothesised as an indicator for susceptibility [13, 21]. In Trichomonad parasites, agreement between beta-tubulin sequences and susceptibility to benzimidazoles *in vitro* has been established for *T. vaginalis* [20, 21]. However, a study on *H. meleagridis* found that while histomonal amino acid sequences predicted a susceptibility to benzimidazoles, no correlation was found with *in vitro* activity for these agents [16].

The aim of this study was to test the *in vitro* activity of albendazole, flubendazole, mebendazole, nocodazole, triclabendazole and thiabendazole against clinical isolates of *D. fragilis* and to determine whether beta-tubulin sequences can be used as an indicator for benzimidazole susceptibility in protozoa.

## Materials and methods

### Parasite culture

Four strains of *D. fragilis* were isolated and propagated *in vitro* using a biphasic xenic culture system using a Loeffler’s slope medium modified from a previously published method [6] consisting of an inspissated horse serum slope overlaid with 5 mL of PBS and supplemented with 2–5 mg of rice starch.

### Genotyping of *D. fragilis* strains

Genotyping was performed as previously described targeting the SSU rRNA gene [30].

### Antimicrobial agents and susceptibility testing

The following antimicrobial agents were used in susceptibility testing: albendazole, flubendazole, mebendazole, nocodazole, triclabendazole and thiabendazole (Sigma-Aldrich, Australia). All benzimidazoles were supplied in powdered form and dissolved in dimethylsulfoxide (DMSO) to make stock solutions of 5 mg/mL. Further doubling dilutions (PBS) were prepared from 1,000 μg/mL to 4 μg/mL. The respective dilutions were added to the PBS overlay at a 1:1 ratio to a final volume of 5 mL, giving a final dilution range of 500 μg/mL to 2 μg/mL of antimicrobial agent in the media. All susceptibility testing was performed in triplicate. A control consisting of 1 mL of 10% DMSO diluted (PBS) into a total of 5 mL and then doubling dilutions were performed (in triplicate) for all drugs to rule out inhibitory effects of DMSO on *D. fragilis*.

The cell concentrations were determined using Kova slides viewed under phase-contrast microscopy at a magnification of X400. Susceptibility testing with each compound was performed over 4 days. Minimum lethal concentrations (MLCs) were determined to be the concentration of the drug at which no trophozoites were observed. A control consisting of a benzimidazole sensitive strain of *Trichomonas vaginalis* (isolated from a local clinical sample) was used to ascertain efficacy of the antimicrobial agents tested (albendazole, flubendazole, mebendazole, nocodazole, triclabendazole and thiabendazole) as previously described [37]. A positive control was also included consisting of the *D. fragilis* cells and the reference drug metronidazole (Sigma Aldrich, Australia) as previously described [24].

### RNA extraction for molecular analysis

Two of the four isolates of *Dientamoeba* used in the susceptibility testing experiments underwent further molecular testing. Ribonucleic acid was extracted from culture sediments using TRIsure reagent (Bioline, catalogue number BIO-38032) and enriched for eukaryotic mRNA using oligo (dT)-cellulose chromatography. Sequencing of the transcriptome was performed by the service provider AGRF (http://agrf.org.au/). The methods used to sequence and assemble the transcriptome of *D. fragilis* will be published elsewhere.

### Mining the transcriptome for tubulin sequences

Contigs from the *D. fragilis* transcriptome were used to construct a blast database using the makeblastdb program available from the NCBI website. *Histomonas meleagridis* beta-tubulin 1, (GenBank accession no.: AEN84279) was used as a query sequence in a tblastn search (default parameters, version 2.2.28+) against this database to identify homologues within the *D. fragilis* transcriptome. Putative *D. fragilis* beta-tubulin sequences detected in this blast search were then subjected to blastn and blastx searches against the NCBI nucleotide and protein databases, respectively, to confirm their identity. Putative *D. fragilis* beta-tubulins were translated into their protein sequences using the “Translate” component of the “Sequence manipulation suite” (Stothard 2000) (website: http://www.bioinformatics.org/sms2/translate.html). Alignments of the resulting amino acid sequences were performed using clustalW (default parameters).

## Results

### Genotyping

All four *D. fragilis* strains used in the experiments were identified as genotype 1.

### MLCs

All benzimidazoles tested (albendazole, flubendazole, mebendazole, nocodazole, triclabendazole and thiabendazole) had no effect on the *in vitro D. fragilis* cultures with MLCs of >500 μg/mL. Metronidazole, however, was effective with an MLC of 31 μg/mL. The *T. vaginalis* control strain was susceptible to all benzimidazoles with MLCs ranging from 4 to 16 μg/mL. Thus, the observed lack of activity against *D. fragilis* is not due to benzimidazole degradation at any point during the experiment.

### Identification of beta-tubulin transcripts in the *D. fragilis* transcriptome

Three *D. fragilis* contigs from *D. fragilis* isolate 1 were identified as close homologues of *H. meleagridis* beta-tubulin (GenBank accession no.: AEN84279) by tblastn search. However, only two of these could be translated into a full length tubulin amino acid sequence. The two full length tubulin contigs achieved significant blastn and blastx hits to beta-tubulin sequences from other trichomonads when blasted against the NCBI web server, confirming that at least two beta-tubulin isoforms are present in *D. fragilis*. These two *D. fragilis* beta-tubulin sequences can be found in GenBank under accession nos. KM186141 and KM186142.

### Examination for amino acids predictive of albendazole susceptibility

**Figure 1.**
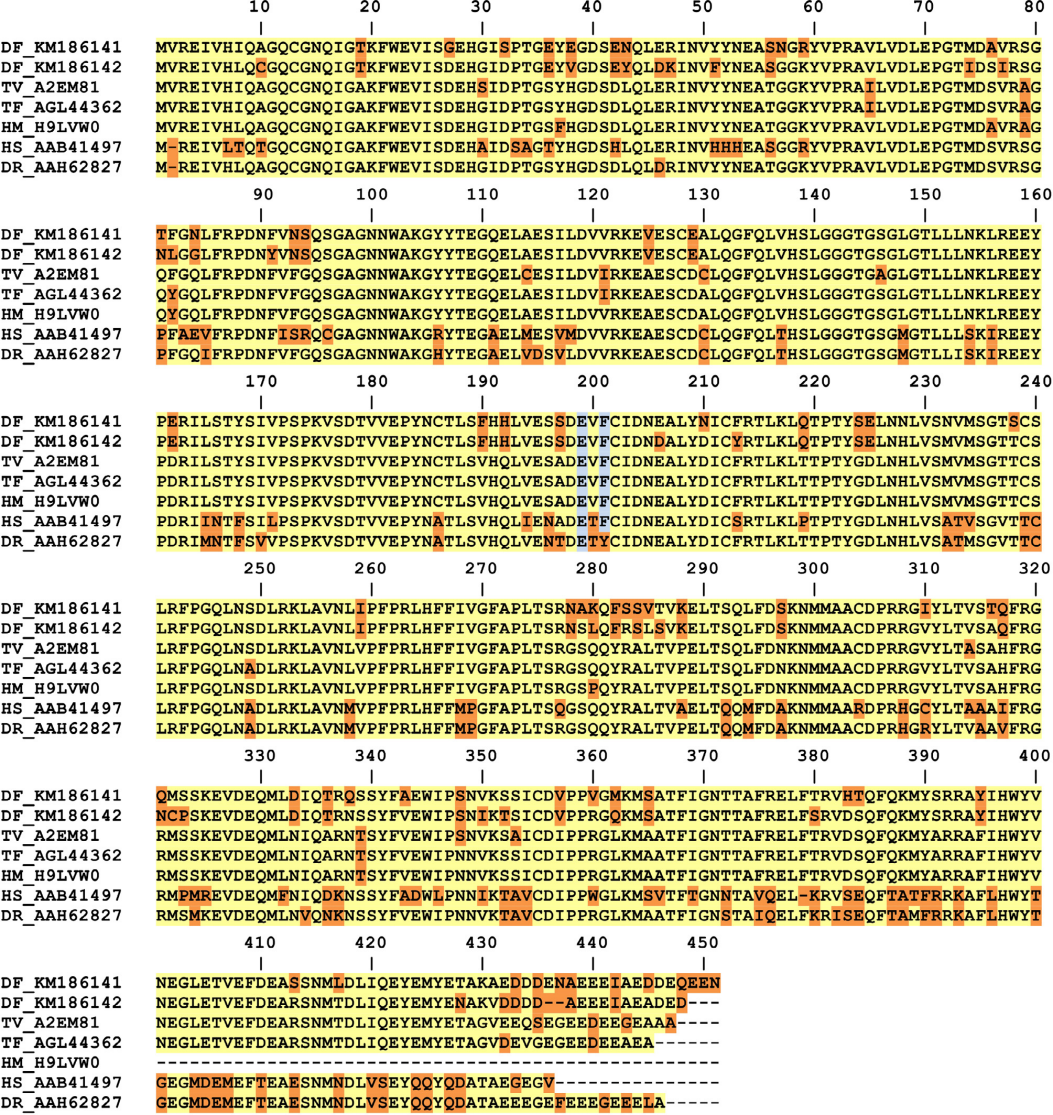
Full alignment of beta-tubulin amino acid sequences from *D. fragilis* with tubulin sequences derived from Trichomonads and other eukaryotes. The residues highlighted blue are those thought to be predictors of albendazole susceptibility in protozoa as described in previous studies. Amino acids shaded yellow represent the most common amino acid at that position (predicted consensus based on this alignment). Amino acids shaded orange are those which differ from the predicted consensus. Note however that at positions 8, 430, 434 and 446, a consensus cannot be resolved. TV: *Trichomonas vaginalis*, DF: *Dientamoeba fragilis*, HM: *Histomonas meleagridis*, TF: *Tritrichomonas foetus*, HS: *Homo sapiens*, DR: *Danio rerio*. For *Histomonas meleagridis* and *Trichomonas vaginalis* the species acronym is followed by the corresponding UniprotKB identifier. For all other organisms, the species acronym is followed by the corresponding Genbank accession number.

Alignment of *D. fragilis* amino acid sequences of beta-tubulin 1 and 2 to beta-tubulins from other Trichomonads (Fig. 1) confirmed that *D. fragilis* possesses the amino acids which are predictive of albendazole susceptibility. Based on these alignments, it became apparent that Trichomonad beta-tubulins possess an additional valine residue which follows the first methionine amino acid. This valine residue was not present in other beta-tubulin sequences examined (such as *Candida sp.*, *Aspergillus sp.* and *Ascaris sp* – data not shown) and the implications of this are that the amino acids predictive for albendazole susceptibility are moved forward by one additional position (see Fig. 1), compared to previous reports describing beta-tubulin sequences [16, 21].

Based on the results of the current study, amino acid positions 198 (199 for Trichomonads) and 200 (201 for Trichomonads) cannot be used as predictors of albendazole resistance (or susceptibility). We suggest, therefore, that there may be other amino acids in the beta-tubulin protein which may be predictive of albendazole susceptibility in protozoa. Alternatively, it may be that the beta-tubulin sequence alone cannot be used as a reliable predictor for albendazole resistance (or susceptibility) in protozoa.

## Discussion

*Dientamoeba* is a frequently encountered enteric protozoan, yet despite the relatively high prevalence of this organism [2, 27], very little research has been undertaken on susceptibility testing to drugs. There is no gold standard treatment for *D. fragilis*, and the majority of treatment data is based on a small number of case reports [26]. Many cases of treatment failure have been reported [4, 28, 36] leading some researchers to postulate that current treatment options may be suboptimal for the eradication of *Dientamoeba* [26]. This highlights the need for further study on antiprotozoal agents that have potential activity against *D. fragilis*. While *Dientamoeba* can be readily cultured from fresh un-refrigerated clinical samples, long-term cultures have been shown to be notoriously difficult to maintain [23]. This has hampered many *in vitro* studies of this organism in particular susceptibility testing. However, recent advances in culturing techniques have allowed for long-term subculture of isolates [6, 23].

Current data is lacking on susceptibility profiles for *D. fragilis* isolates with only three previous studies conducted to date [3, 10, 25]. Only two of these used clinical samples, with one using the no longer available *D. fragilis* ATCC strain 30948 which was of the rarely encountered genotype 2 type, which is not the predominant genotype found in clinical samples [30]. The current study used four clinical isolates of *D. fragilis*, all of which were genotype 1.

Benzimidazoles have been widely used since the 1960s as anthelmintic agents in veterinary and human medicine and as antifungal agents in agriculture. Initially, benzimidazole activity seemed to be limited to helminths and fungi however in 1985 *T. vaginalis* was reported to be inhibited by the benzimidazole derivatives mebendazole and flubendazole [19]. Subsequently, susceptibility of benzimidazoles was shown for *G. intestinalis* and microsporidia [21]. More recently, the activity of benzimidazoles was tested against *H. meleagridis* and they were shown to be an ineffective agent for treatment *in vitro* [16]. Resistance to the benzimidazoles has been observed in parasitic nematodes of livestock animals since the early 1960s [11]. The beta-tubulin protein confers benzimidazole sensitivity in the helminth *Caenorhabditis elegans* and clear evidence exists that three different single amino acid substitutions (Thr-167, Glu-198 and Phe-200) in the beta-tubulin protein of different nematode species can be responsible, each leading separately to resistance [8]. However in protists, it seems that only two may play a role, namely Glu-198 and Phe-200 [21].

Although the complete crystallographic structure of the beta-tubulin monomer and the mechanism of action of benzimidazoles are still unknown, a recent study used homology modelling techniques along with molecular docking studies to advance this area of research [1]. The study was undertaken on *Trichinella spiralis* and the researchers were able to suggest a binding site for benzimidazoles that contains several amino acids associated with resistance (Phe-167, Glu-198 and Phe-200). This further supports the role of these amino acid positions in albendazole resistance or susceptibility in helminths.

The current study used several benzimidazole derivatives: albendazole, flubendazole, mebendazole, nocodazole, triclabendazole and thiabendazole. All were shown to be ineffective anti-*Dientamoeba* agents. Concentrations ranging from 2 μg/mL to 500 μg/mL resulted in *D. fragilis* trophozoite cell counts similar to that of the control. Although both *Giardia* and *Trichomonas* have been shown to be susceptible to benzimidazoles, the closely related *H. meleagridis* was shown to be resistant to benzimidazoles [9, 16, 17]. The exact mechanism for resistance is however unknown [16].

Based on this study, positions 198 and 200 of the beta-tubulin protein are not predictive of albendazole resistance, indicating that we need to look elsewhere to understand the phenomenon of resistance to benzimidazoles in Trichomonads. It should also be noted that this phenomena has not only been reported in Trichomonads. *Giardia* strains can reportedly become resistant to albendazole without having mutations in Glu-198 or Phe-200 [34]. Taken with the results of the current study, this detracts from the importance of Glu-198 and Phe-200 in albendazole susceptibility as seen in protozoa. Clearly, other mechanisms of albendazole resistance must be explored in protozoa.

## Conclusion

The results of this study show that benzimidazoles have no effect on *D. fragilis* in culture. As such, no therapeutic response could be expected from the treatment of dientamoebiasis with benzimidazoles. The preliminary data presented would also suggest that beta-tubulin sequences cannot be used as a reliable marker for resistance of benzimidazoles in *D. fragilis* and as a result, other markers of benzimidazole resistance need to be explored.
